# Viral lysis accelerates microbial succession patterns resembling diatom senescence

**DOI:** 10.1093/ismejo/wrag206

**Published:** 2026-08-05

**Authors:** Timotej Turk Dermastia, Ion Gutiérrez-Aguirre, Tinkara Rupnik, Tinkara Tinta

**Affiliations:** National Institute of Biology, Marine Biology Station Piran, Fornače 41, Piran 6330, Slovenia; National Institute of Biology, Department of Biotechnology and Systems Biology, Večna pot 121, Ljubljana 1000, Slovenia; University of Ljubljana, Department of Microbiology, Večna pot 111, Ljubljana 1000, Slovenia; National Institute of Biology, Marine Biology Station Piran, Fornače 41, Piran 6330, Slovenia

**Keywords:** diatoms, virus, bacteria, *Pseudo-nitzschia,* shunt, senescence, microbiome, phycosphere

## Abstract

Diatom blooms influence carbon cycling through organic matter production and its deposition or remineralization—processes mediated by the microbial community. Viruses can influence diatom bloom dynamics and even terminate blooms, yet interactions between diatoms, their viruses, and associated bacteria remain poorly resolved. Here, we examined how infection of the toxigenic diatom *Pseudo-nitzschia galaxiae* by its ssRNA virus PnGalRNAV reshapes host physiology, microbiome structure, and organic-matter processing in non-axenic batch cultures. Using epi-fluorescence microscopy, 16S rRNA amplicon sequencing, and metatranscriptomics, we linked microbial composition, localisation, and functional activity during viral lysis. Infection rapidly collapsed diatom growth and induced a senescence-like host state, with broad repression of photosynthesis, silicon metabolism, and core biosynthetic pathways, alongside induction of heat-shock and other stress-related genes. Concurrently, phycosphere-associated bacteria declined, detritosphere-associated bacteria increased, and community composition shifted from *Marinobacter* (*Gammaproteobacteria*) dominated, towards *Flavobacteriaceae* (*Bacteroidetes*) dominated, especially by *Polaribacter*. In non-infected controls *Alphaproteobacteria* proved to benefit from the stable healthy phycospheres with a distinct DOM pool. Bacterial metatranscriptomes showed significant upregulation of polysaccharide-degradation-associated genes in infected cultures, indicating active utilisation of lysis-derived diatom glycans. Similar compositional and metabolic profiles in infected cultures and later-stage senescent controls suggest infection accelerated senescence-associated microbial processes. Overall, viral lysis converted a productive diatom culture into a detrital, DOM-rich environment that selects for specialized polysaccharide degraders, redirects carbon through the viral shunt and may accelerate nutrient recycling in coastal systems. Extending this approach to natural microbial communities and diverse diatom–virus systems will help determine whether these mechanisms are broadly conserved.

## Introduction

Phytoplankton, as a major eukaryotic, unicellular group, depend on the supporting bacterial community for growth and survival [1]. The interactions between them occur in the phycosphere, the immediate surroundings of phytoplankton cells, where nutrient and chemical exchange between phytoplankton and bacteria takes place [2]. Diatoms are important constituents of marine phytoplankton communities and are not exempt from interactions with bacteria [3–5]. Various *Bacteroidetes, Gammaproteobacteria,* and *Alphaproteobacteria* are often associated with diatom blooms and in cultures [3, 6–9]. Diatoms (and phytoplankton alike) are also affected by viruses. Viruses are an integral part of marine microbial communities [10]. They outnumber bacterial cells by a factor of 10 and unicellular eukaryotes by a factor of 10^4^ [11]. Viruses infecting phytoplankton primarily disturb the production of biomass by killing their hosts and transforming the particulate organic matter (POM) to dissolved organic matter (DOM) fuelling the microbial loop, a process also known as the “viral shunt” [12]. This process also leaves a distinct metabolic fingerprint in the derived DOM [13, 14]. Recent data also show that DOM derived from different viruses infecting the same host, elicits a very different response in the microbial growth [15]. On the other hand, viruses can also enhance carbon export in certain conditions, which is known as the “viral shuttle” [16]. This process mainly unfolds through the increase of aggregating potential of lysed or infected cells because of the production of sticky exudates [17–19]. Aggregation can also be influenced by bacteria and the ways they digest or transform organic matter produced by infected or dying cells [20]. The interplay between phytoplankton, bacteria, and viruses is however not well studied. Bacteria readily exploit the DOM released from lysed cells but may also have other indirect effects on phytoplankton. It has been shown for example that bacteria may confer resistance to certain viruses in *Chaetoceros tenuissiumus* cocultures [5]. This finding adds to the understanding that phytoplankton have associated microbiomes that boost their fitness. In our study we focused on the diatom *Pseudo-nitzschia galaxiae*, a toxigenic member of the genus whose microbiome was not studied before. Microbiomes associated with other *Pseudo-nitzschia* species consist of host-specific bacterial communities whose composition and diversity vary with host species and toxicity, reflecting co-adaptation and a continuum of interactions from mutualistic to parasitic. These bacterial communities are often dominated by taxa affiliated with Gammaproteobacteria (e.g. *Marinobacter, Alteromonas, Pseudoalteromonas, Pseudomonas*), Alphaproteobacteria (e.g. *Phaeobacter*), and *Bacilli* [21, 22]. In this study we exposed a culture of *P. galaxiae* to its lytic virus PnGalRNAV to better understand the response of the microbial community from the diatom culture to DOM released by lysed diatom cells as well as to the changed physiological state of infected but still living cells. Here, we also define senescence as a state of the diatom culture where decline due to nutrient stress or bottle effects occur despite the presence of a virus. During our experiment we monitored abundance of diatoms and specific bacterial populations via fluorescence in situ hybridization using epifluorescence microscopy, and virus abundance via qPCR, concentration of inorganic nutrients and DOM. We applied omic approaches (16S rRNA gene amplicon sequencing and metatranscriptomics) to study the dynamics of microbial community structure and function.

## Materials and methods

### Experimental setup

The experimental setup was previously described [23]. Briefly, a non-axenic culture of *Pseudo-nitzschia galaxiae* (isolate Pn208, 28S accession number OR482970) was grown in a 10-litre Duran flask containing 8 L of L1 medium with silicate. The culture was partitioned into 6 1-litre flasks representing 3 biological replicates of infected (~ 1:100 virus:diatom ratio) and 3 non-infected cultures. At each time point, we subsampled 60 mL of the batch cultures, from which we devoted aliquots for enumeration of diatom [23], bacterial and virus abundance, and measurement of inorganic and organic nutrient concentrations. Throughout the manuscript, sampling time is reported as experimental day (Day X), where Day 0 denotes virus addition to infected cultures and parallel handling of controls. Subsamples for 16S rRNA amplicon sequencing (200 mL onto 0.22 μm polycarbonate filters) and metatranscriptomics (500 mL initially and varying volumes later onto 1.2 μm cellulose filters) were collected from the initial batch culture and on Day 5. On Day 5, ~700 mL of culture remained prior to filtration for DNA, well within the recommended ⅔ of the initial volume for representativeness, and ~500 mL remained after filtration for nucleic acids. We continued the experiment despite reduced representativeness and subsampled again on Day 9 for all parameters except nutrients and DOC. All filters for omic analysis were instantly frozen and stored at −80°C until further analysed.

### Diatom and bacterial counts

**Figure 1:**
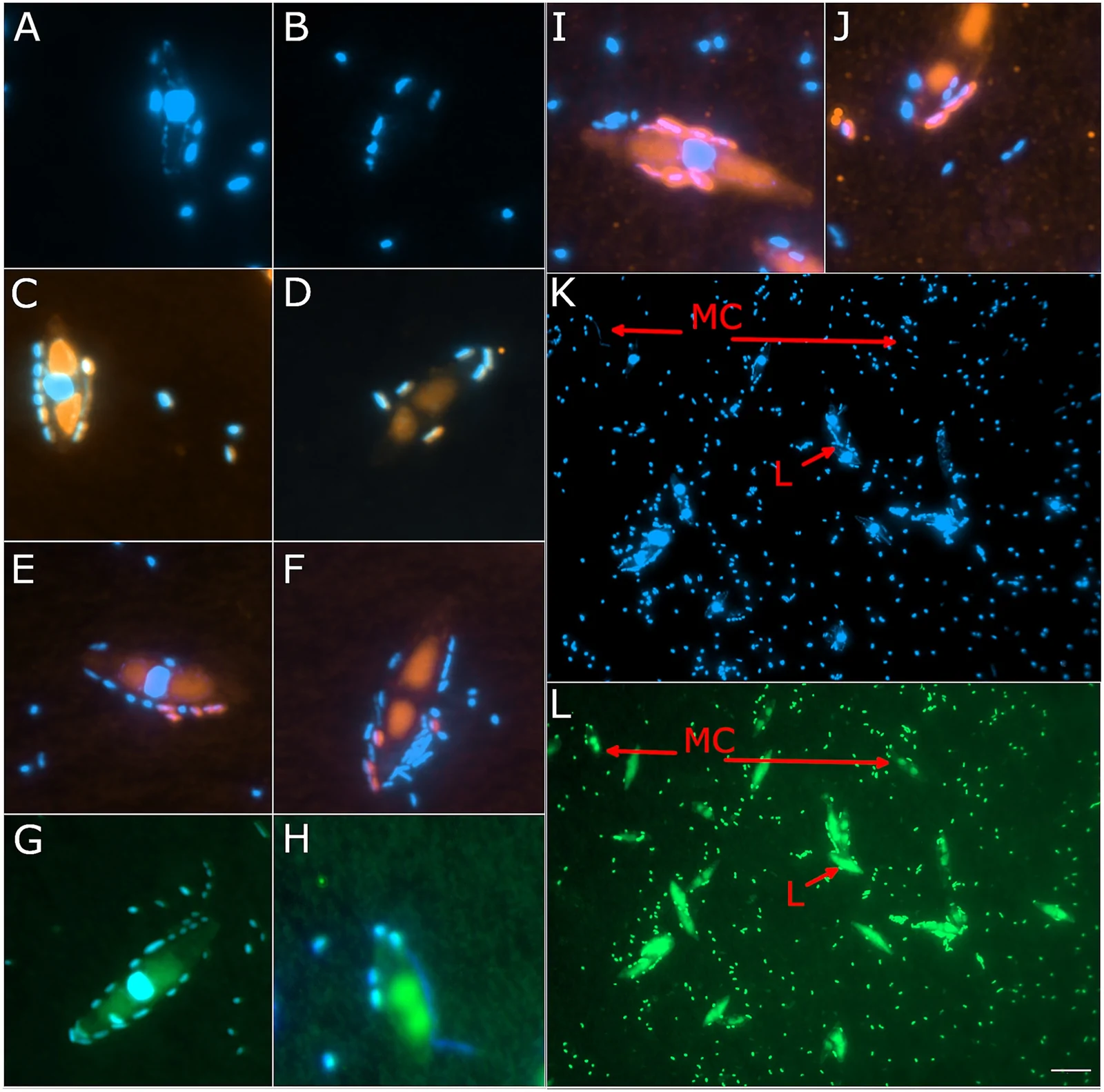
Epifluorescence microscopy determination of compromised/living cells and the phyco- and detrito-spheres with selected fluorochromes and FISH probes. A,B) DAPI stain; C,D); *bacteria* probes (EUB I, II, III) with DAPI; E,F); *Alphaproteobacteria* probe (Alf968) with DAPI; G,H); *Bacteroidetes* probe (CF319a) with DAPI; I,J) *Gammaproteobacteria* probe (Gamma42a) with DAPI. DAPI (K) and *Bacteroidetes* FISH probe (L) of infected diatoms 5 days post infection. Membrane compromised diatoms (MC) are identified by a missing DAPI stain but visible signal with the other probe. In this case chloroplasts are clearly visible. Bacteria attached to such cells were counted as detritosphere associated. In contrast, living cells (L-arrow) have clearly stained nuclei with DAPI. Bacterial cells attached to such cells were counted as phycosphere associated.

Diatoms were enumerated as previously described [23]. At the same time, 1 mL of sample was fixed with 2% formaldehyde (final concentration) and stored at −80°C until further processed: filtered onto 0.2 μm white polycarbonate filter, mounted with DAPI (Ex/Em = 358/461 nm) and examined with epifluorescence microscopy at 1250× magnification for bacterial abundance and diatom identification, as previously described [24]. We partitioned the bacterial counts into free-living bacteria and diatom attached bacteria (phycosphere- and detritosphere-associated). Intact diatom cells were identified by the presence of an intact DAPI-stained nucleus and two chloroplasts (Fig. 1A, C, E, G, I, K, L). In addition, we quantified diatom detritus or detritosphere–associated bacteria, corresponding to bacteria attached to membrane-compromised or lysed diatom cells, which were identified by a visible frustule or by probe-stained chloroplasts in the absence of a DAPI-stained nucleus (Fig. 1B, D, F, H, J, K, L). This definition also corresponded to our identification and enumeration of membrane-compromised diatom cells. DAPI stains DNA and does not directly assess viability; however, the combined presence of an intact nucleus and chloroplasts is a good indication of structurally intact (presumably living) cells. In addition, cells may be non-viable while still retaining an intact nucleus, and therefore our classification reflects structural integrity of membranes rather than strict viability. Diatoms were counted at all time points, while bacterial abundance was determined on Days 0, 3, 5, and 9.

### Fluorescence in situ hybridization

The abundance of specific bacterial populations was determined by fluorescence in situ hybridization (FISH) using specific oligonucleotide probes labelled with fluorophore Cy3 or FITC at the 5′-end (Biomers) (Table S1). The specific target bacterial populations were selected based on the relative abundances of bacteria from 16S rRNA gene amplicon sequencing data (top 3 most abundant bacterial classes were selected). We applied a modified version of the FISH method as before [25].

### Free virus quantification

1 mL of 0.22 μm filtrate was obtained at each time point by syringe filtration (Millex) of culture subsamples and was used to extract RNA according to the protocol described previously [23]. To quantify the virus, we developed a new TaqMan qPCR assay, targeting a 135 bp long amplicon in the RdRP gene within the viral genome (forward primer: GAAGAGGATGAAAAGGAAG; reverse primer: GAGTCCAGACATAACAACA, TaqMan probe: [FAM]AAAGTTGCGGAAAAGAGCAGA[TAM]) as a complement to the previously published assay targeting the capsid gene [23]. Assay optimization and testing followed MIQE guidelines [26]. The assay is described in more detail in Supplementary Method 1.

### DOM and nutrient measurements.

Samples for dissolved organic carbon (DOC) and inorganic nutrients were measured as previously [24]. Briefly, samples were filtered onto combusted Whatman GF/F filters (∼0.8 μm, 25 mm) and stored at −20°C until analysis. DOC was measured using high-temperature catalytic oxidation (Shimadzu TOC-L with TN unit), calibrated with potassium phthalate and potassium nitrate, and validated with certified reference materials (Hansell Lab). Reproducibility was ≤2%. Dissolved inorganic nutrients were determined spectrophotometrically using segmented flow analysis (QuAAtro, Seal Analytical) following standard methods, with accuracy verified using reference materials (KANSO) and regular intercalibration (QUASIMEME) [24].

### DNA and RNA extraction and sequencing

DNA from bacterial samples was extracted using the DNeasy PowerWater Kit (Qiagen, Hilden) following the manufacturers guidelines (Table S2). The V4-V5 16S region was amplified using primers 515-Y and 926R [27]. Library preparation and sequencing on a HiSeq System (Illumina) using 2x250 bp sequencing with a depth of 100 000 tags per sample was performed by Novogene Ltd (Cambridge, UK).

Filters for RNA extraction, including a blank filter for negative control of extraction, were submerged in TriZol reagent (Zymno Research), 0.1 mm and 0.5 mm bashing beads were added (ZR BashingBead, Zymno Research), and the mixture was vortexed vigorously for a minute. The mixture was then flash frozen, thawed, and vortexed again for a minute. This was repeated 3 times, before proceeding with extraction according to the manufacturer’s protocol using the Direct-zol RNA Miniprep kit with DNAse treatment (Zymno Research). The RNA concentration and integrity was measured with a HS RNA assay on the Tapestation (Agilent) (Table S2). Sequencing was performed by Novogene Ltd. on a NovaSeq X Plus System (Illumina) using a 2x150bp configuration following rRNA depletion. Negative extraction filters failed QC and were not sequenced.

### 16S rRNA amplicon analysis

The outputs from 16S rRNA amplicon sequencing were, quality-checked, merged, and trimmed using the R (v4.5.1 [28]) package *DADA2* [29] standard pipeline and compared to the SILVA 99% 16S rRNA gene database (v138) for community composition inference. Alpha and beta diversity analyses were conducted in *phyloseq (v1.52)* [30] and *vegan (2.7.3)* [31] packages. ANOSIM based on Bray–Curtis distances was used to test overall and pairwise differences in community composition among groups, and *DESeq2* (v1.38.2) [32] was used for statistical testing of group based and taxon based differences, respectively.

### Bacterial metratranscriptomes

Metatranscriptomic reads were quality-checked and adapter-trimmed using *CLC Genomics Workbench* (QIAGEN) with default parameters for RNA-Seq workflows. High-quality paired-end reads were then assembled de novo using *rnaSPAdes* (v3.15.4), optimized for transcriptome reconstruction without a reference genome [33]. The assembled transcriptome was translated using *TransDecoder* (v5.7.1) (https://github.com/TransDecoder/TransDecoder) and the resulting peptides were clustered at 98% using CD-HIT. The top transcript in each cluster was used as a reference index for read mapping. Transcript-level abundance was quantified per sample using *salmon* (v1.9.0) in quasi-mapping mode [34]. For functional and taxonomic annotation, the full transcriptome was run through *eggNOG-mapper* (v2.1.12) [35]. Additional annotation was performed using blast on an in-house database of the target virus genomes, to map unannotated contigs to the PnGalRNAV genome. To estimate the completeness of microbial carbohydrate utilization pathways, peptides obtained with *TransDecoder* on the entire set of assembled transcripts, were further annotated with dbCAN3 [36].

### Host transcriptomes

We analysed the diatom host transcriptomic response to viral infection using a reference-based approach leveraging publicly available *Pseudo-nitzschia* transcriptomes from the MMETSP project [37]. These transcriptomes were concatenated and dereplicated using *CD-HIT* [38] and functionally annotated with dammit, which integrates tools including *TransDecoder*, *hmmer* (v3.4) [39]*,* and *diamond* (v2.1.11) [40]. Transcript quantification was performed using s*almon*, aligning metatranscriptomic reads from each sample to the annotated reference transcriptome.

### Differential expression analysis

*edgeR* (v4.0.16) [41] was used for normalization and subsequent differential expression analysis (DEA) with sensitivity tests run in *ALDEx2* (v1.30.0) [42] and *DESeq2 (v1.48.1)* [32]. Low abundance contigs were filtered out with *filterByExpr*, using experimental condition as the grouping variable, and further filtered manually by retaining only contigs with >10 counts in at least four samples. This additional filtering step was guided by quality-control inspection of median log2 counts per million (CPM) distributions, which substantially stabilized variance in the bacterial dataset, although the effect was less pronounced in the diatom dataset. Library-size and compositional biases were normalized using the trimmed mean of M-values method (TMM), applying *TMMwsp* to bacterial data and *TMM* to diatom (host) data. Differential expression was analysed separately for bacterial and diatom transcript sets using quasi-likelihood negative binomial generalized linear models implemented in *edgeR*. Quasi-likelihood models were fitted with glmQLFit using robust estimation, and differential expression was tested with glmQLFTest. Genes were considered differentially expressed (DE) based on a false discovery rate < 0.05 and an absolute log2 fold change > 1. See Supplementary Method 2 for parameterization of ALDEx2 and DESeq2 functions and additional analyses.

### Statistical analysis

Where reported, data were analysed using a linear model with treatment day, and their interaction as fixed effects. Model assumptions were assessed using residual diagnostics. Pairwise comparisons between treatments were performed using estimated marginal means (*emmeans* v2.0.3 [43]) with Tukey-adjusted *P* values to obtain model-based treatment means adjusted for the other terms in the model within each day.

## Results

### Viral attack, diatom population collapse, and organic matter release

**Figure 2:**
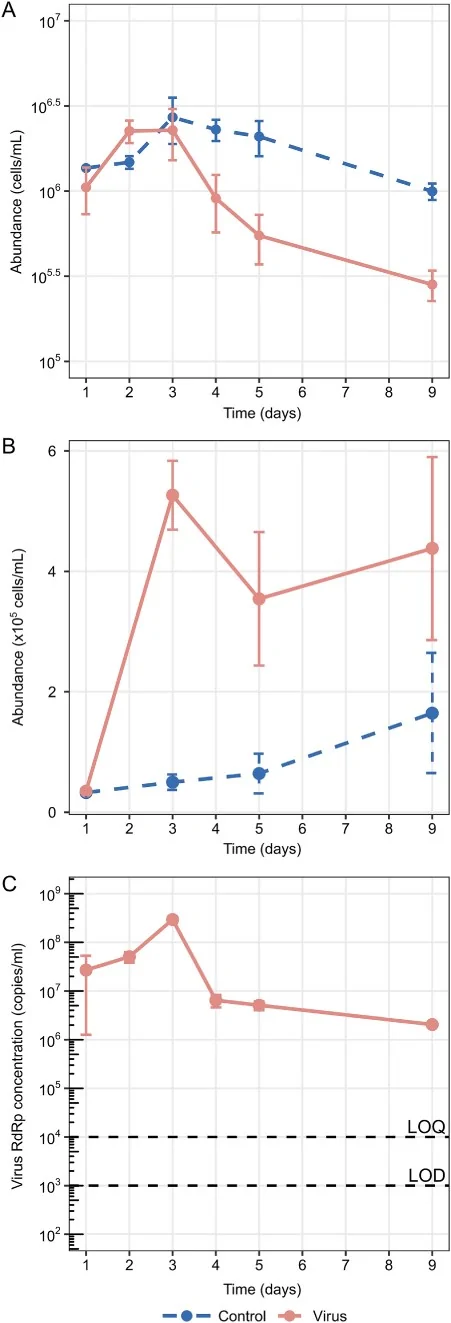
Dynamic of diatoms and free viruses throughout the experiment. Living diatoms (A) adapted from [23] with permission (reprinted from Harmful Algae Turk Dermastia et al., discovery of novel and known viruses associated with toxigenic and non-toxigenic bloom forming diatoms from the northern Adriatic Sea, 102 737, 2024, with permission from Elsevier, licence no. 6285270017915). Diatoms with compromised membranes (B) as detailed by the absence of the DAPI stained nucleus and simultaneous FISH-staining of chloroplasts or clear visibility of the frustule. C) Free virus concentration obtained by the novel qPCR assay targeting the RdRP gene of the PnGalRNAV. Control samples in the qPCR assay were all below the LOQ as well as bellow the LOD and are therefore considered without virus (see Supplementary Method 1 for all Cq values of samples and calibration curve and LOD/LOQ determination. Error bars are standard errors. Number of biological replicates for each group and day =3. Linear model and marginal means estimation: ^*^ (*P* < .05); ^**^ (*P* < .001).

In a previous study [23], we confirmed that both the control and virus treatments showed an initial increase in diatom cell numbers toward Day 3 of the experiment, followed by a quick collapse of the culture in virus-infected treatments, and a more gradual decrease in cell numbers in the non-infected controls (Fig. 2A). The collapse occurred once the diatom population had reached the stationary phase in both treatments, but it was delayed by 5 days in the control treatment. Concurrently, diatoms with compromised membranes increased in both treatments, but the change was much more pronounced in infected treatments (Fig. 2B), especially after Day 3. Later there appeared to be a slight drop in the abundance of such diatoms. The viral copy number followed the pattern of diatom population dynamics and decreased about 10-fold to initial inoculum values as the diatom culture collapsed (Fig. 2C, Table S3). However, 10^6^ copies/mL of the viral RdRP gene remained detected after this collapse. Virus-treated cultures visibly discoloured by Day 5, consistent with rapid lysis, while controls retained the brown pigmentation of healthy diatom cultures. (Fig. S1).

The DOC concentration increased almost 2-fold from the start of the experiment onwards in virus-infected cultures and 1.5-fold in controls, as both the control and infected cultures started to decline (Fig. S2). DOC accumulation in infected cultures became significantly greater than in controls from Day 4 onwards (estimate = −66.17, *P* = .0175), potentially reflecting increased organic matter release due to diatom lysis. DOC was highest on Day 5 in two infected flasks, reaching 586 μM and 583 μM in Virus1 and Virus2, respectively (estimate = −88.92, *P* = .0024). The diatoms in both infected and control cultures depleted nitrate, silicate, and phosphate without significant differences between treatments (Fig. S3). On the other hand, there was a stronger buildup of ammonium in the infected treatments (Fig. S3). The difference was not overall statistically significant, but estimated marginal means showed a significant difference on Day 5 (estimate = −3.61, *P* = .0016).

### Dynamics of the microbial community

#### Structure of microbial community based on amplicon sequencing of bacterial 16S rRNA genes

**Figure 3:**
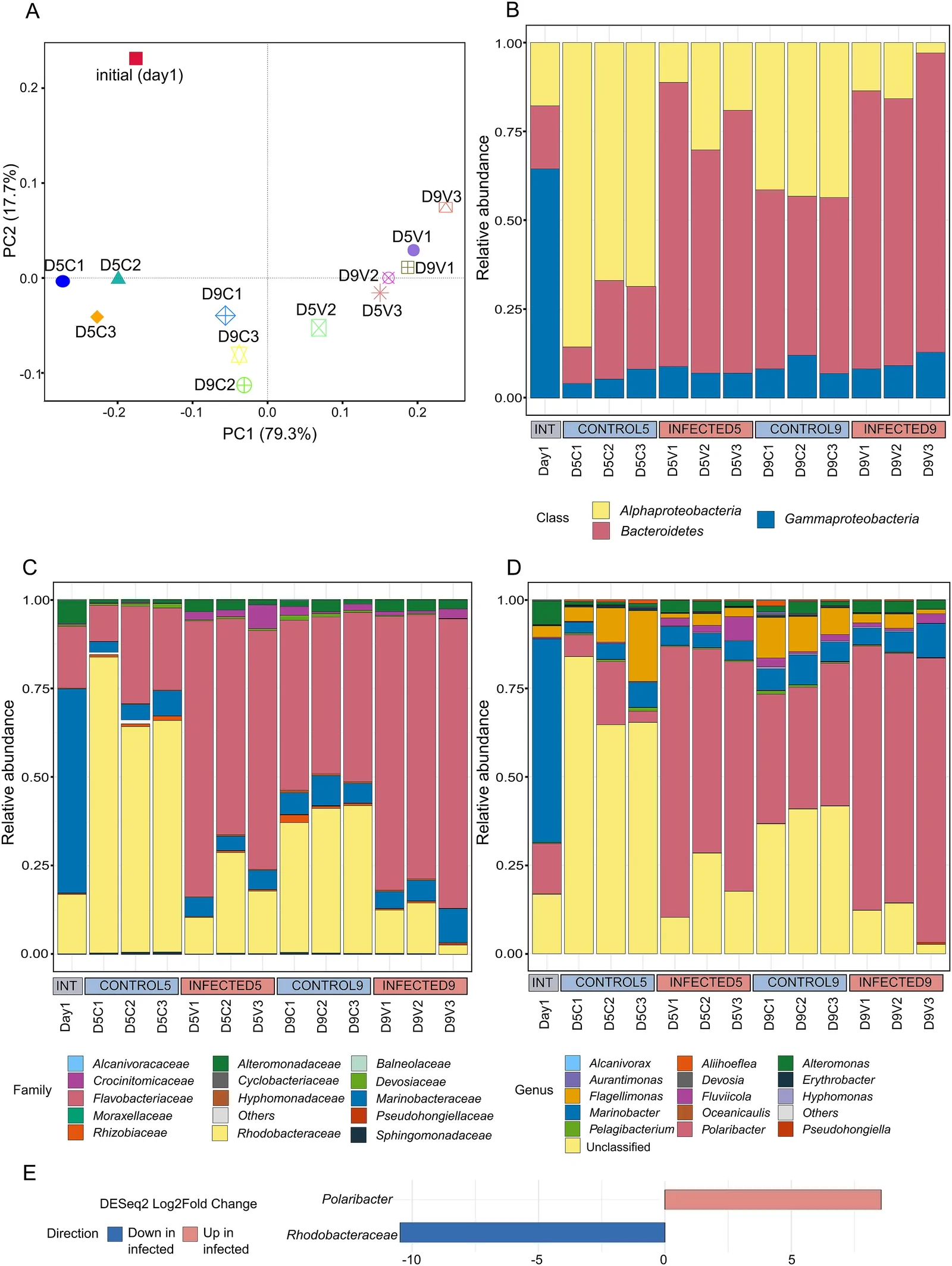
Bacterial genus taxonomy inferred from 16S rRNA amplicon sequencing. See supplementary figs S5 for complete representation. (A). PCoA with weighted unifrac distances. (B) Relative abundances of bacterial classes. (C) Relative abundances of top 15 bacterial families. (D) Relative abundances of top 15 bacterial genera. (E) Significantly altered taxa from DESEq2 analysis and their log2fold changes between controls and infected cultures at day 5. Init-initial condition at Day0; control5 – Non-infected samples on Day5; control9 – Non-infected samples on Day9; infected5 – Infected samples on Day5; infected9 – Infected samples on Day9.

Amplicon sequencing detected 333 ASVs, with low diversity across treatments (Shannon Index ≤2.5; species richness ~25, Fig. S4). The initial bacterial community in the exponential growth phase consisted mainly of *Marinobacter* (~60%) and *Alteromonas* (both Gammaproteobacteria), and of lower proportions of *Polaribacter* (~20%) and *Flagelimonas/Muricauda* (both *Bacteroidetes*). Principal coordinate analysis (PCoA) showed strong separation of initial samples from both treatments, and convergence of control with infected samples, indicating that senescent controls shifted toward an infected-like community state (Fig. 3A). ANOSIM supported significant overall group differences (R = 0.85, *P = .*0003) despite non-significant pairwise tests. Both treatments exhibited strong temporal restructuring. *Marinobacter* declined sharply from early dominance to low abundance (~5%) by Day 5 (Fig. 3D), while *Bacteroidetes*, primarily *Polaribacter*, expanded from 20% to ~70% relative abundance in infected cultures and to ~50% in controls (Fig. 3B). Control flasks also showed increases (80% relative abundance) in unclassified *Rhodobacteriaceae* (*Alphaproteobacteria*) on Day 5 (Fig. 3C, Supplementary Figs S4A and S4B). Manual inspection with BLAST against the NCBI database of the most abundant ASVs belonging to this group revealed that these were likely uncultured *Roseovarius* bacteria. DESeq2 analysis confirmed significant enrichment of *Polaribacter* in infected treatments and *Rhodobacteriaceae* in controls (Fig. 3E, Table S4).

#### Population dynamics of specific microbial populations via microscopy-based approach

**Figure 4:**
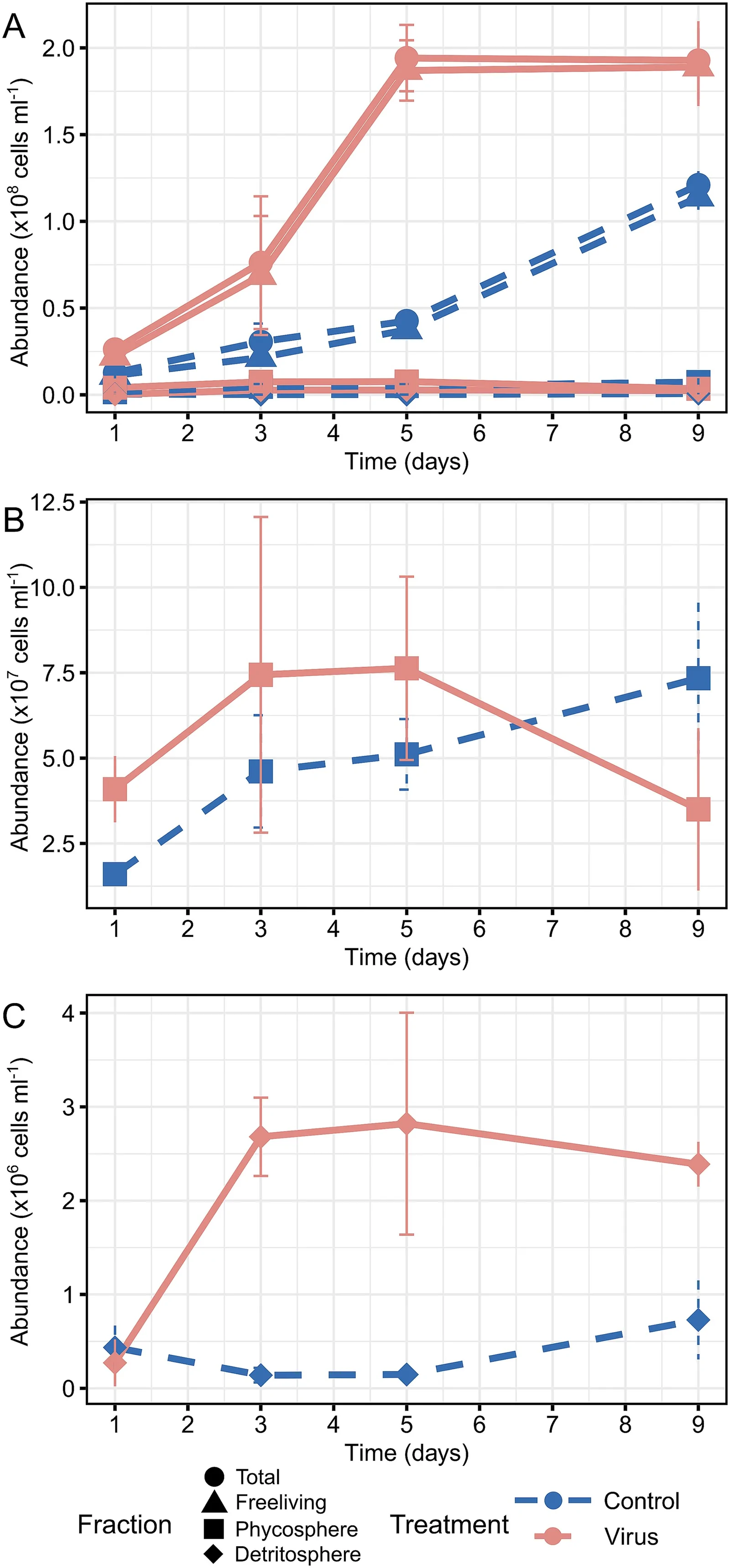
Dynamics of the microbial population from DAPI staining. All fractions (A). Close up for scaling of phycosphere associated microbes (B), and detritosphere associated microbes (C). Error bars are standard errors. Linear model and marginal means estimation: ^*^ (*P* < .05); ^**^ (*P* < .001).

Total microbial abundance (DAPI-stained cells) rose tenfold by Day 5 and remained stable through Day 9 in virus treatments (Fig. 4A), while reaching ~1.5-fold lower abundances by Day 9 in controls. *Bacteria* (EUB-positive cells) constituted on average ~90% of microbial community in all treatments (Table S5). Phycosphere-associated microbes showed similar initial abundance in both treatments; they increased steadily in controls (~4.6-fold by Day 9) but in infected treatments rose modestly to Day 5 (~1.8-fold) before declining as diatoms collapsed (Fig. 4B). Detritosphere-associated microbes increased profoundly in the infected treatments, while remaining constant in controls (Fig. 4C). Bacteria per uncompromised diatom (phycosphere) increased from 1.3 ± 0.45 (control) and 4.7 ± 6.23 (infected) on Day 1 to 2.75 ± 1.15 (control) and 29.16 ± 18.84 (infected) by Day 5, then declined in infected cultures (Fig. S5A). Attached bacteria per compromised diatom (detritosphere) in controls declined from 13.27 ± 12.37 to 2.8 ± 2.7 by Day 3 and then stabilized (Fig. S5B), whereas detritosphere microbial loads in infected treatments remained stable at ~5–8 bacteria per cell.

**Figure 5:**
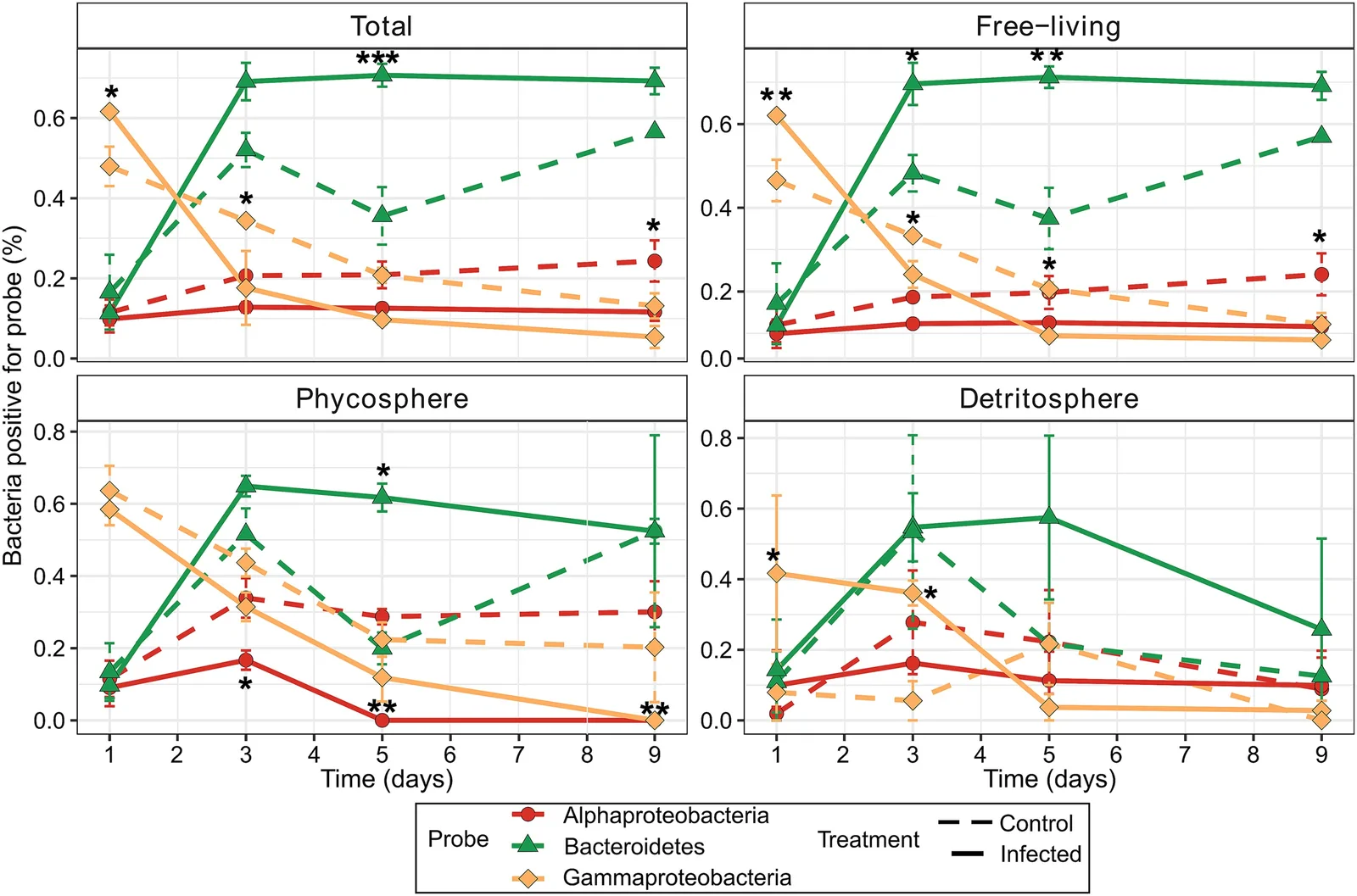
Dynamics of specific bacterial populations (*Alphaproteobacteria, Gammaproteobacteria,* and *Bacteroidetes*) within total microbial community, free-living population, phycosphere- and detritosphere-associated microbial community in control vs virus treatments over time. Linear model and marginal means estimation: ^*^ (*P* < .05); ^**^ (*P* < .001); ^***^ (*P* < .0001).

*Bacteroidetes* drove most of the total microbial community increase in both treatments (Fig. 5). In infected treatments, their relative abundance rose ~6.5-fold by Day 3 and thereafter dominated the total microbial community (~70% of DAPI-stained cells), while they reached only ~56% in the control by Day 9. During the experiment, *Alphaproteobacteria* increased more in controls (~1.9-fold), whereas *Gammaproteobacteria* declined in both treatments, but the decrease was 1.7-fold greater in infected treatments as compared to controls (from ~45% to ~13% in controls and 63% to ~9% in infected). Free-living fractions showed comparable trends to the total bacterial community.

*Bacteroidetes* also dominated the phycosphere (Fig. 5) in infected treatments after Day 3, reaching ~52% by Day 9, whereas in controls they declined after Day 3 to ~20% and increased again to ~50% by Day 9. *Gammaproteobacteria* continuously decreased through the experiment in both treatments until Day 9 but were consistently higher in control than in infected treatments, with 1.7-fold difference at the end of the experiment. The difference was even more pronounced for *Alphaproteobacteria*, which were ~ 2-fold more abundant in the phycosphere of control treatments by Day 3 and remained stable at ~20% thereafter, whereas they were completely depleted in infected cultures. Detritosphere shifts were more pronounced: *Bacteroidetes* reached ~57% of detritosphere-associated microbes in infected treatments by Day 5, followed by a decline down to 26% by Day 9. Instead, in control treatments *Bacteroidetes* started to decline already after Day 3 down to ~12% by Day 9. *Gammaproteobacteria* dropped from ~42% to ~0.07% in infected cultures, while they increased to ~21% by Day 5, followed by a complete decline by Day 9 in the control. *Alphaproteobacteria* showed a transient peak on Day 3 in the detritosphere (~16%) in infected treatments before declining, with a similar dynamic, but slightly higher peak in controls (~27%).

### Metatranscriptome sequencing results

Sequencing generated ~80-100 M reads per sample. On Day 5, viral RNA dominated infected samples (90% of reads), limiting host and microbial insights (Table S6). Bacterial reads comprised 60 ± 6.7% in control cultures on Day 5 versus 6.2 ± 1.2% in infected cultures on Day 5.

#### Broad expression patterns

**Figure 6:**
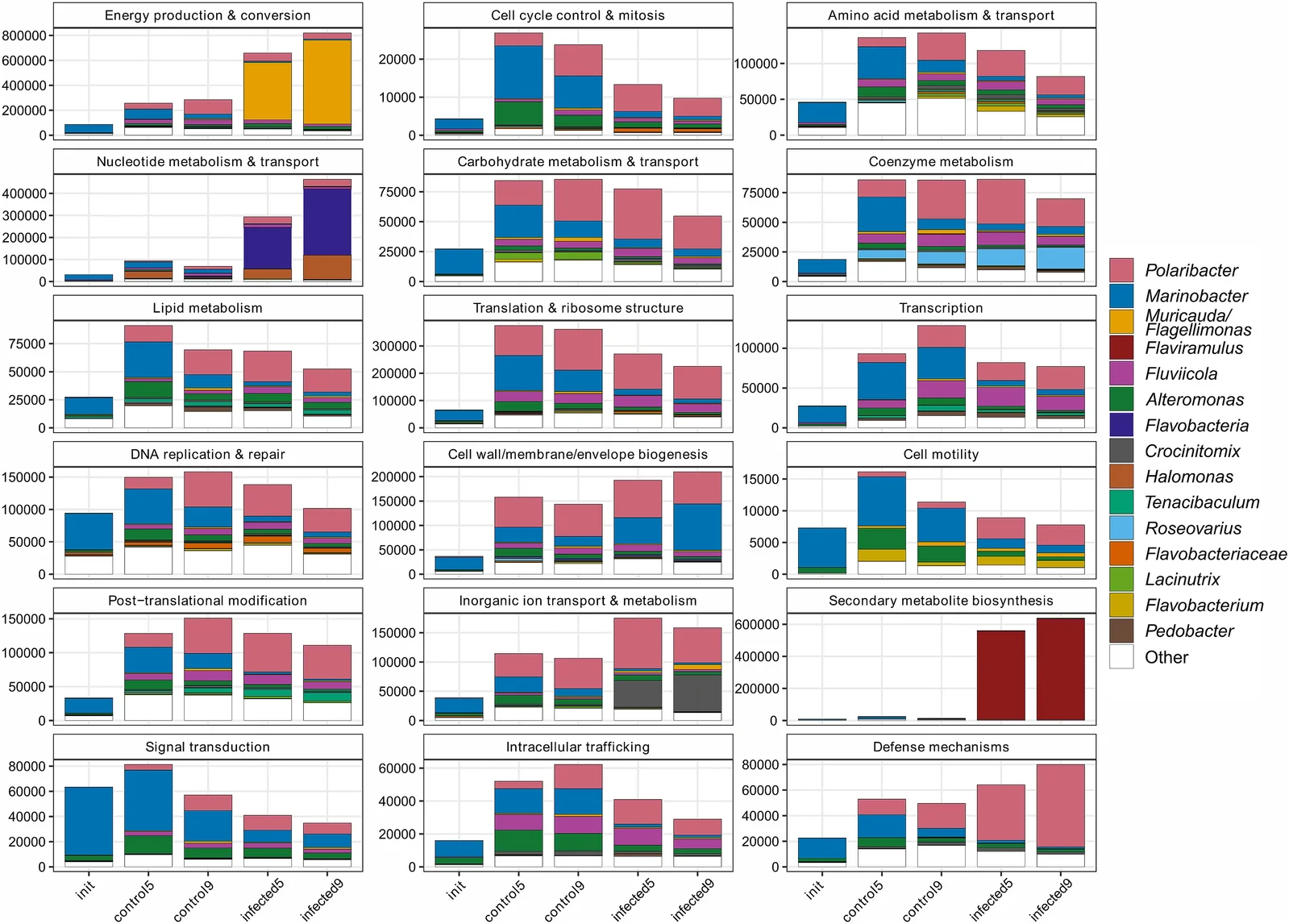
Counts per million (TMM-normalized) of genes belonging to different COG categories (eggnog annotation) between genera. Init-initial condition; control5 – Non-infected samples on Day5; control9 – Non-infected samples on Day9; infected5 – Infected samples on Day5; infected9 – Infected samples on Day9. All abundances are average TMM across three biological replicates.

After TMM normalization, COG categories showed marked CPM differences and taxonomic shifts (Fig. 6). Transcriptional activity increased in both treatments, but key metabolic processes shifted from being primarily conducted by *Marinobacter* to *Flavobacteriaceae* (especially *Polaribacter*). The bacterial community in infected cultures showed increased expression of carbohydrate metabolism genes, but this was not clear from COG G (carbohydrate metabolism and transport) alone, because some carbohydrate degradation genes were annotated to other COG groups. For example, highly expressed *Flaviramulus* secondary metabolite genes (annotated as COG Q alginate lyases) and *Crocinitomix* inorganic ion transport genes (annotated as COG P *TonB*-dependent receptors) linked to carbohydrate metabolism, both added to the expression levels of carbohydrate metabolism genes. Other clusters of orthologous genes (COGs) with higher expression in infected cultures included energy production (COG C, *Muricauda* ~ 3× higher) and nucleotide metabolism (purine and pyrimidine synthesis in *Flavobacteriia* and *Halomonas,* respectively; ~3 and ~ 7× higher in Day 5 and Day9, respectively). Conversely, the bacterial community in infected cultures showed a decreased expression of motility (~2-fold decrease), signal transduction (~2-fold decrease), and a ~ 30–40% decrease in growth-associated genes (translation, transcription). The large relative abundance of *Rhodobacteriaceae* in control samples on Day 5 revealed by 16S rRNA amplicon sequencing was not evident here, although *Roseovarius* did represent a notable portion of coenzyme metabolism, but with stable expression in both treatments. Overall, although functions were conserved, expression levels and specialist taxa contributions varied substantially across treatments.

#### DE bacterial COGs

**Figure 7:**
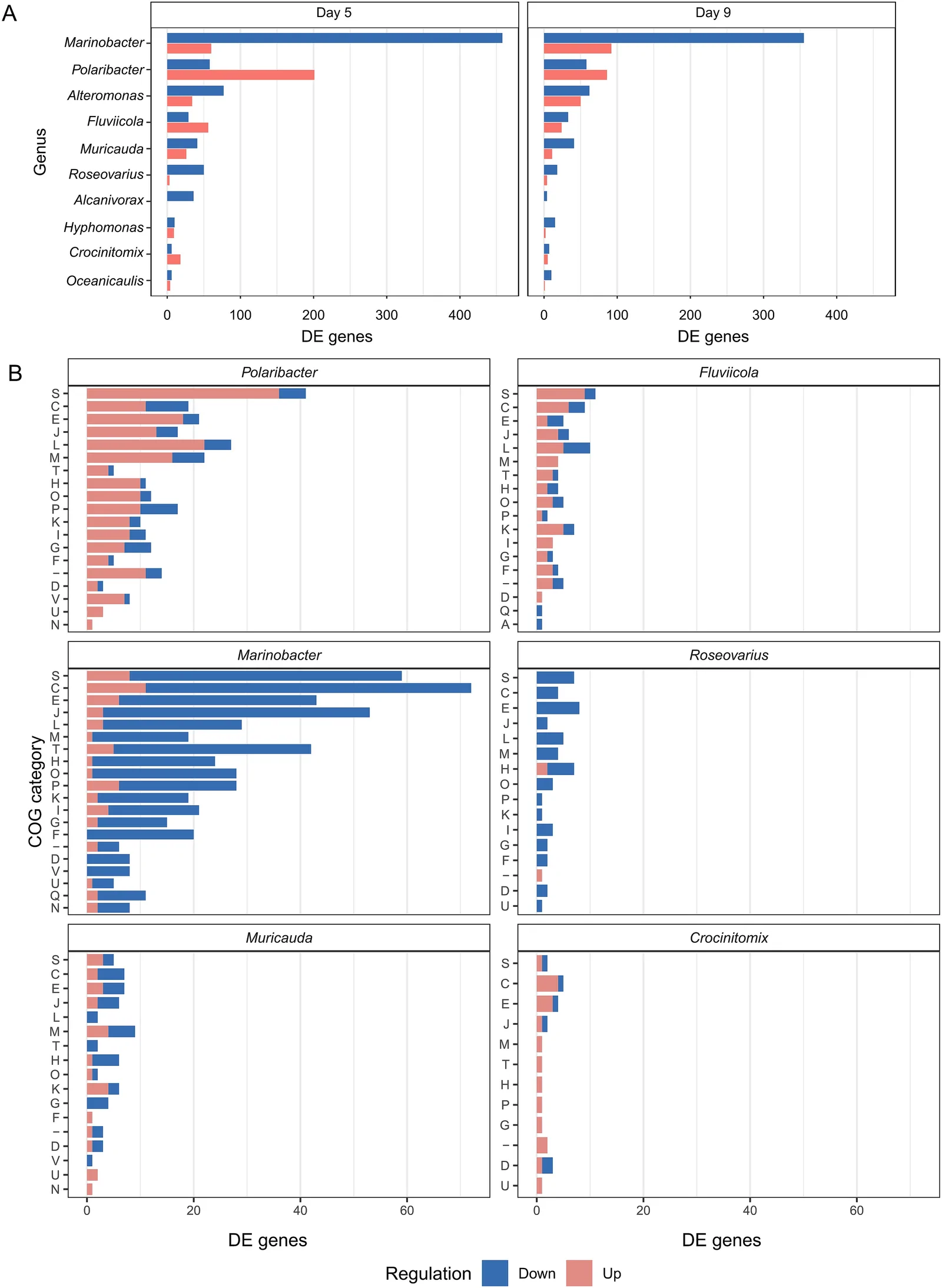
Results of DEA. The number of DE genes in the top 10 genera by abundance for each day of the experiment (A). COG categories of DE genes for day 5 DEA (B). The baseline for expression were control (non-infected) samples. Regulation direction is for infected samples. COG categories are: A = RNA processing and modification; C = energy production and conversion; D = cell cycle control, cell division, chromosome partitioning; E = amino acid transport and metabolism; F = nucleotide metabolism & transport; G = carbohydrate metabolism & transport; H = coenzyme metabolism; I = lipid metabolism; J = translation & ribosome structure; K = transcription; L = DNA replication & repair; M = cell wall/membrane/envelope biogenesis; N = cell motility; O = post-translational modification; P = inorganic ion transport & metabolism; Q = secondary metabolite biosynthesis; S = function unknown; T = signal transduction; U = intracellular trafficking; V = defence mechanisms; W = extracellular structures; “-“= unannotated.

Normalization through the *edgeR* pipeline yielded the most comparable DE datasets while accounting for compositional differences between infected and non-infected samples. Most downregulated genes belonged to *Marinobacter*, and their sheer number made the infected community appear less transcriptionally active overall due to high *Marinobacter* expression in controls (Fig. 7A). Some *Marinobacter* genes were also heavily upregulated in infected cultures, such as a pilus forming protein, linked to cell attachment on Day 5. Otherwise, upregulated genes were largely associated with *Polaribacter*, *Fluviicola*, and *Crocinitomix*—all *Flavobacteriia*—on Day 5 (Table S7A), and exclusively with *Polaribacter* on Day 9 (Table S7B). These upregulated genes spanned diverse COG categories, including cell motility in *Polaribacter* and *Muricauda*, and numerous COGs in *Crocinitomix* (Fig. 7B). Consistent with bacterial processing of organic nitrogen, upregulated COG E genes included peptidases, peptide/amino-acid transporters, aminotransferases, gamma-glutamyltransferase, serine dehydratase, and arginine-metabolism genes, although the broader downregulated COG E signal was largely associated with the decline of *Marinobacter*. A similar but delayed and less pronounced metabolic response occurred in control cultures on Day 9 relative to Day 5, with broad upregulation of *Polaribacter* and downregulation of *Marinobacter* COG groups (Fig. S6), pointing to a similar, but delayed microbiome restructuring in control cultures on Day 9, comparable to infected cultures on Day 5. *Alphaproteobacteria*, specifically *Rhodobacterales* such as *Roseovarius* and *Roseobacter* had more upregulated genes in controls, especially those related to amino-acid metabolism, membrane generation, and energy production/conversion (Table S7). There were more upregulated DE genes in controls on Day 5 (Fig. 7B) than on Day 9 (Fig. S6) but the pattern was similar to infected cultures.

#### DE CAZymes and carbohydrate metabolism genes

Roughly 1000 contigs carried CAZy annotations. Infected cultures on Day 5 showed slightly more upregulated CAZymes than controls, again driven by heavy *Marinobacter* downregulation and upregulation in *Polaribacter*, *Fluviicola*, and other *Flavobacteriia* (Fig. S7). By Day 9, DE CAZy genes were fewer and shifted toward downregulation, with the previously upregulated genera showing diminished activation. Among CAZy families, infected cultures on Day 5 uniquely upregulated several hydrolytic families: polysaccharide lyases (e.g. PL6 alginate lyase), several glycoside hydrolases, and carbohydrate-binding modules. On Day 9 only some glycoside hydrolases and carbohydrate-binding modules remained upregulated.

Transcripts with large positive fold changes included *TonB* receptors associated with complex carbohydrate uptake. On Day 5, *Polaribacter* showed distinct coexpression clusters linking TonB/SusC-like receptors with upregulated PL6 and glycoside hydrolase transcripts in infected treatments, plus a smaller downregulated *susC*-containing cluster (Fig. S8A). By Day 9, this structure was largely retained, but the PL6-associated cluster disappeared, and fewer genes passed the correlation threshold (Fig. S8B). In *Marinobacter*, the Day 9 network was reduced (Fig. S8D) compared to Day 5 (Fig. S8C), with fewer correlated genes and loss of differential expression in most receptor-associated nodes, despite persistent downregulation of associated CAZyme transcripts in infected cultures.

Substrate profiling showed that CAZyme expression was highly genus- and substrate-specific. β-glucan-associated genes were among the most abundant overall and alginate degradation by *Flaviramulus* was particularly pronounced in infected samples. Day 9 was characterized more broadly by higher expression of glucan-degrading genes in non-infected cultures, with some clear genus-specific exceptions; by contrast, *Crocinitomix* β-mannan/chitin degradation and *Alteromonas* (e.g. *Crocinotomix, Zobellia*) pectin degradation remained comparatively consistent across conditions (Table S8, Fig. S9).

### Host-transcriptome

**Figure 8:**
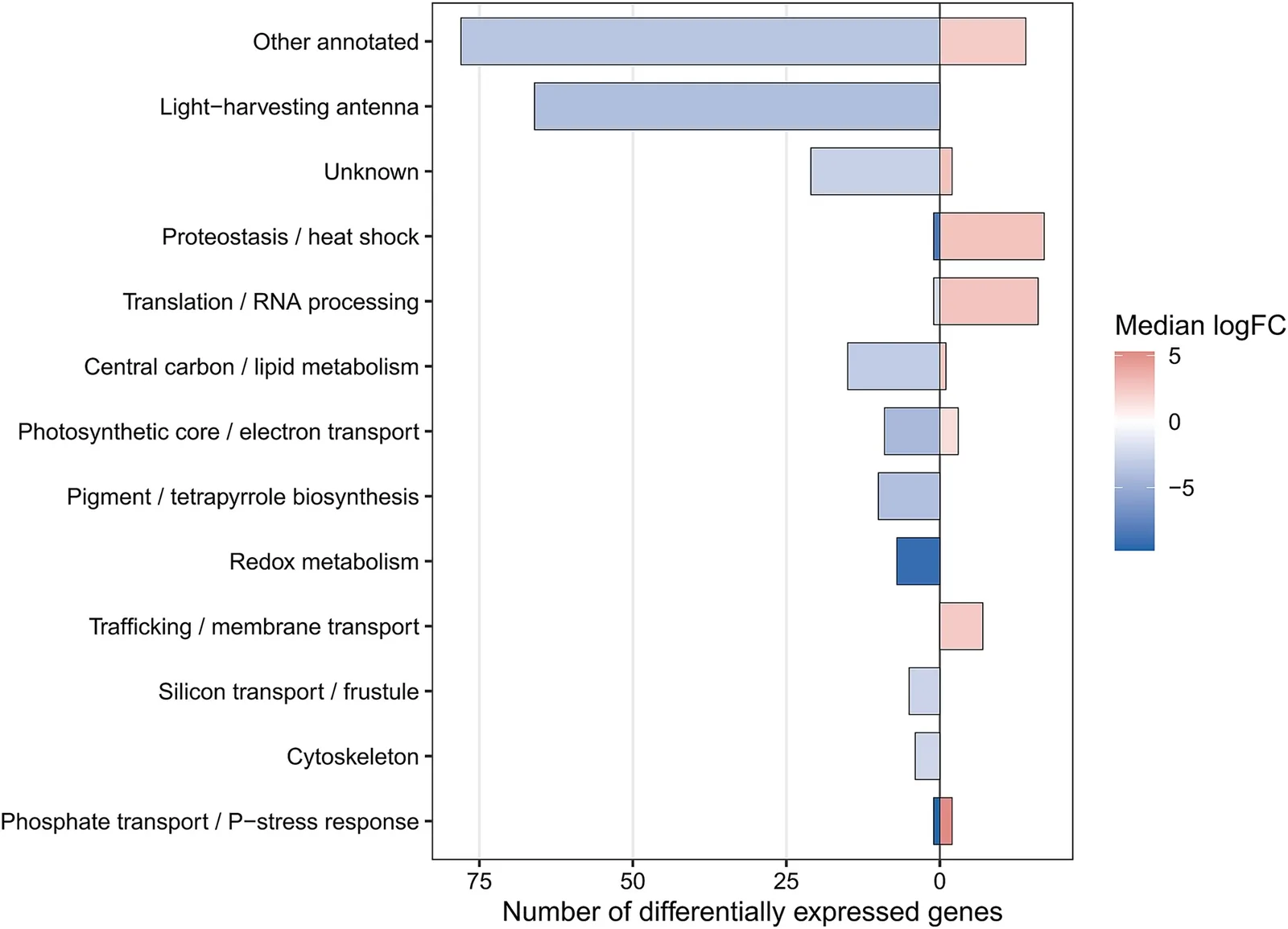
Significantly DE genes of the host on day 5 of the experiment between infected and non-infected cultures. Non-infected samples were used as the baseline. Right side of the plot indicates downregulation; left side of the graph indicated upregulation in infected samples. The colour hue depends on median log_2_ fold change in expression of all genes in the group relative to the baseline.

The overall representation of diatom transcripts was very low in infected treatments (below 1% of all transcripts, Table S5). This could be attributed to transcriptomic shutdown as well as the competition of viral RNA for sequencing depth. However, data normalization in *edgeR* did allow for a comparison of a set of genes that were present in all of the samples. Median expression of the tested gene set was roughly similar between samples, except for Day 9 where only one replicate had representable expression (Fig. S10). The DEA comparing infected and control cultures on Day 5 resulted in 218 downregulated and 62 upregulated genes in the infected samples. We detected a pronounced downregulation of genes associated with the photosynthetic apparatus, mainly light-harvesting antenna proteins and photosynthetic core proteins (Fig. 8, Table S9A). Central metabolic pathways also collapsed during infection as evidenced by pronounced downregulation of oxidoreductases. Silicon transport, a key component of diatom frustule construction was also predictably reduced. However, the transcriptome did not collapse across the board, and some important functions were retained. These were associated with processes pointing to cell hijacking by the virus, such as increased translation and RNA processing, potentially functioning as machinery to produce new viruses, which was also evidenced by pronounced upregulation of Na/P transporters consistent with heavy phosphorus demand. Lastly, there was strong upregulation of some heat shock proteins, which may reflect both a stress response to infection and correction of protein folding in dying cells. When the DE analysis was conducted on Day 9 samples, most of the differences present on Day5 were not detected (Table S9B), although the set of DE genes was smaller (50 downregulated, 8 upregulated in infected samples). This comparison was not as robust however, with only one infected sample having comparable normalized expression levels to control samples.

## Discussion

### Viral takeover and accelerated senescence of the diatom host

This study examined the compositional and functional response of the diatom associated microbiome to viral infection of its host. In the *Pseudo-nitzschia galaxiae*–PnGalRNAV type-II system, viral infection leads to rapid collapse of diatom culture [23]. Free virus copy numbers, estimated by two different qPCR assays [23], exhibited an unusual trend of decline from ~10^7^–10^8^ mL^−1^ to 10^6^, despite dominance of viral transcripts, likely reflecting the removal of viruses entrapped in progressively accumulating cellular debris or extracellular matrix, during the filtration step. Because metatranscriptomics was done on Days 5 and 9, when most cells were already lysing, our analysis captured the late stages of infection rather than the onset of host responses. By Day 9, diatoms in both treatments had reached advanced physiological decline, displaying similar profiles of bacterial community structure and function, whereas not many differences on the host-transcriptome level were detected. This convergence indicates that ssRNA viral infection can compress normal senescence into a rapid lytic event followed by bacterial growth and organic matter recycling. This pattern is consistent with the viral shunt, in which lysis redirects POM into dissolved pools accessible to heterotrophic bacteria [44–46]. Infected cultures showed stronger nutrient depletion and higher ammonium accumulation than controls, consistent with increased bacterial degradation of protein-rich organic matter during lysis [47]. Transcriptomic data partly supported this interpretation, although the sampled bacterial community had already reached stationary phase, suggesting a scavenging-oriented response rather than active growth. In addition, nitrogen recycling responses varied among bacterial taxa rather than reflecting uniform activation of ammonification pathways.

### Bacterial exploitation of viral lysis products

Viral infection induced lysis of *Pseudo-nitzschia* cells and release of organic matter, reflected by the increase of DOC concentrations (Fig. S2). At the same time, 1.5-fold higher increase of microbial community abundance was determined in infected cultures (Fig. 4). Although microbiomes in long-term cultures are generally stable [48], PnGalRNAV-mediated lysis altered resident microbial community structure and the relative importance of individual taxa.

The decline of phycosphere-associated microbes in infected cultures after Day 5 suggests that infection destabilized interactions among living diatom and bacteria. Our results suggest that newly infected or lysed cells initially acted as hotspots for bacterial attachment, evident from the increase of detritosphere-associated microbes after Day 1 in the infected treatment. However, after Day 5 we observed a decline of detritosphere-associated microbes and a concurrent rise of microbes in the free-living fraction. In contrast, phycosphere-associated microbes continuously increased in control treatments, while detritosphere-associated microbes increased only slightly towards the end of the experiment and remained at abundances approximately twofold lower than in the infected treatment. As infection progressed, our preliminary microscopy-based observations suggest that aggregates became less compact and were replaced by an expanding exopolymer matrix in which bacteria were embedded or formed separate aggregates (Figs 21, 23, 28 in Table S10). This reflects models of marine snow and phytoplankton aggregate microbiology, which emphasize that aggregates act as transient nutrient hotspots subject to intense colonization, rapid turnover, and high rates of detachment driven by particle disaggregation and fluid shear [49]. Moreover, virus-infected and non-infected dead diatoms supported different microbial loads and trajectories, implying that viral lysis modifies not only the physical properties of particles but also the composition and bioavailability of the released DOM. This interpretation aligns with studies showing that phytoplankton viral lysis generates DOM pools that are chemically distinct from healthy-diatom derived DOM and that these differences feed back onto bacterial succession, substrate use, and carbon cycling on and around aggregates [15, 20, 50]. Lastly, our results indicated that at least in this simplistic, culture-associated microbial community, natural senescence led to similar structural and functional shifts as with viral infection, which has not been previously reported [45].

*Bacteroidetes* dominated the phycosphere in the exponential and stationary phases of growth of both infected and non-infected diatoms and replaced the predominant member of the resident community - *Marinobacter* (*Gammaproteobacteria*), in line with previous studies [21, 22, 51]. *Flavobacteriia*, especially *Polaribacter,* showed the strongest response to diatom physiological decline, consistent with their role in degrading algal polysaccharides and detrital DOM [52–55]. In infected cultures*, Bacteroidetes* preferentially proliferated on dead and decaying diatoms and later became major components of the free-living community, matching observations that they bridge attached and free-living niches during decaying phytoplankton blooms [56, 57]. Overall, although *Bacteroidetes* followed similar trajectories in both treatments, viral infection supported a more abundant and more persistent *Bacterioidetes*-dominated detritosphere. By contrast, *Alpha*- and *Gammaproteobacteria*, initially comprising ~10% and ~ 60% of the phycosphere, respectively, were depleted after viral infection, although *Marinobacter* strongly upregulated a gene involved in phytoplankton cell attachment on Day 5, suggesting an induced attachment pathway [58]. Their decline suggests that viral lysis shifted substrates from labile DOM toward more structurally complex detrital material, favouring polysaccharide specialists such as *Bacteroidetes* over copiotrophic *Gammaproteobacteria* [7, 46, 52, 56]. The decrease in *Alphaproteobacteria* is also consistent with their association with more stable phytoplankton interactions, including vitamin supply, redox balancing, and signalling, particularly within the *Roseobacter* lineage [8, 59]. Although similar shifts also occurred in senescing controls, infected cultures remained distinct through alternative degradation pathways, including alginate decomposition by *Flaviramulus* and strong carbohydrate-degradation signatures in *Crocinotomix* and *Muricauda*. In controls, *Alpha*- and *Gammaproteobacteria* persisted as substantial phycosphere components, with *Alphaproteobacteria* remaining stable over time (Fig. 5) and showing amino-acid metabolism signatures consistent with previous observations including their preference for algal exudates over lysates [55]. Together, these patterns suggest that infection altered DOM composition in ways that favored *Bacteroidetes*. In our mixed microbial community, complementary enzymatic capabilities likely allowed coupling between hydrolysis and DOM assimilation: *Polaribacter* dominated carbohydrate active enzymes (CAZyme) expression, with contributions from other *Flavobacteriia* (Fig. 7), consistent with guild-level functional plasticity in flavobacterial CAZyme inventories during algal blooms [53, 57, 60]. Infected samples showed marked upregulation of glycan-uptake machinery on Day 5—particularly *TonB*-dependent transporters and *SusD*-like proteins—relative to controls, in line with the recruitment of these proteins during stationary growth phases which our infected system reached by Day 5 and the use of these systems as proxies for polysaccharide scavenging during blooms [55, 61]. This indicates that expression of *TonB*-dependent transporters may be a more robust marker of bacterial glycan scavenging in high-viral-load contexts (Fig. S9).

The DOM generated in this diatom-virus system supported bacterial growth (Fig. 4; Fig. S2), in contrast to recent work on *Chaetoceros tenuissimus* ssRNA infected by an ssRNA virus, where virus-derived DOM did not sustain growth of *Sphingobacterium* isolates but stimulated their exopeptidase activity [15]. Both systems involve Marnaviridae, but different genera (*Salisharnavirus* in our case versus *Bacillarnavirus* for *C. tenuissimus*). Together, our data suggests that the composition and bioavailability of viral-derived DOM for the bacterial community depend on specific host–virus combinations.

### Host transcriptional reprogramming under viral dominance

Despite the overwhelming abundance of viral RNA, host-derived reads still provided insight into cellular reprogramming. The dominance of viral transcripts (~99% of mapped reads on Day 5) severely limited statistical power but with normalization techniques provided by *edgeR* we were able to produce a representable dataset for day 5 and partially representable one for Day 9. Consistent expression patterns across infected replicates suggest that *Pseudo-nitzschia* cells mounted a stress response to infection. Upregulated transcripts included several heat-shock and stress-related genes indicative of oxidative stress responses like those described in other algal virus systems [62]. In *Emiliania huxleyi* infections, for instance, antioxidant pathways such as glutathione metabolism are induced within 24–48 h [62]. The concurrent increase in genes involved in ribosome synthesis and transcriptional activity may indicate viral hijacking of the host translational machinery—a mechanism common among both DNA and RNA algal viruses [63]. Conversely, transcripts associated with photosynthesis, silicon uptake, and central carbon metabolism were downregulated, reflecting the suppression of growth and biosynthetic activity typical of cells approaching lysis [64]. While the DEA on Day 9 was less robust, due to strong transcriptome suppression and compositional differences it pointed to less DE genes, suggesting a convergence in naturally senescing cells to those infected by the virus in the late stages of infection or death. Together our observations portray a shift from active metabolism toward stress response and translational redirection under viral control.

### Experimental and analytical considerations

Our results highlight the challenges of metatranscriptomic analysis under high viral loads. When viral transcripts dominate sequencing depth, even actively transcribed bacterial genes may appear depleted, and relative normalization methods can generate spurious differential expression. Early-stage sampling would capture infection onset before host RNA degradation and allow a clearer view of antiviral responses and the timing of bacterial activation. Moreover, RNA extraction from 1.2 μm filters captured primarily diatoms and particle-attached bacteria, whereas free-living taxa could be underrepresented, although the density and homogenization of the culture likely minimized this effect. Future experiments should assess the free-living community and disentangle its role in DOM turnover. Finally, the use of a long-term cultured diatom strain with an adapted microbiome may have reduced ecological variability compared to natural assemblages.

### Ecological implications and outlook

Together, our observations indicate that PnGalRNAV infection of *Pseudo-nitzschia galaxiae* restructures the microbiome in ways that partly resemble host senescence but cannot be explained as a simple temporal lag. Viral infection altered succession patterns of dominant bacterial taxa, produced distinct phycosphere and detritosphere trajectories, and recruited distinct genes in the most affected bacterial groups.

Viral lysis released organic substrates that supported bacterial growth and accelerated carbon recycling through the viral shunt, potentially shortening bloom turnover. However, outcomes in diatom–bacteria–virus systems likely depend on viral host range, infection dynamics, and microbial responses, which may determine whether lysis primarily enhances DOM availability or promotes aggregation and export through the viral shuttle [17, 19]. Preliminary observations from another experiment [65], together with the aggregate microscopy presented here (Table S10), suggest that PnGalRNAV-induced decay may favour DOM production over aggregation and generate detrital substrates distinct from natural senescence. Resolving this balance will require separating particulate and DOM fractions and linking organic matter composition to microbial dynamics, activity, aggregation, and particle-size distributions.

Future studies comparing multiple diatom–virus pairs across full infection cycles, combined with metabolomic and physiological measurements, will be needed to determine whether the stress-related transcriptional changes and metabolic shutdown observed here are general features of algal virus infections or system-specific outcomes.

## Supplementary Material

Web_Material_wrag206

## Data Availability

Scripts are available through https://github.com/Timzladen/DIAVIR and ZENODO. 16S rRNA amplicon and RNASeq reads and assemblies are available through the PRJEB97092 accession. For individual sample accessions see Table S2. All supplementary data and additional analysis research data is also available through https://doi.org/10.5281/zenodo.21717367.
