# Supplementary material for: Viral lysis accelerates microbial succession patterns resembling diatom senescence
Source: ISME J. 2026 Aug 5;20(1):wrag206. doi: 10.1093/ismejo/wrag206 (PMC13590890; doi:10.1093/ismejo/wrag206)
Supplement: Supplementary file 1 [file web_material_wrag206.zip › Supplementary Figure 10 - Diatom edger QC.pdf]

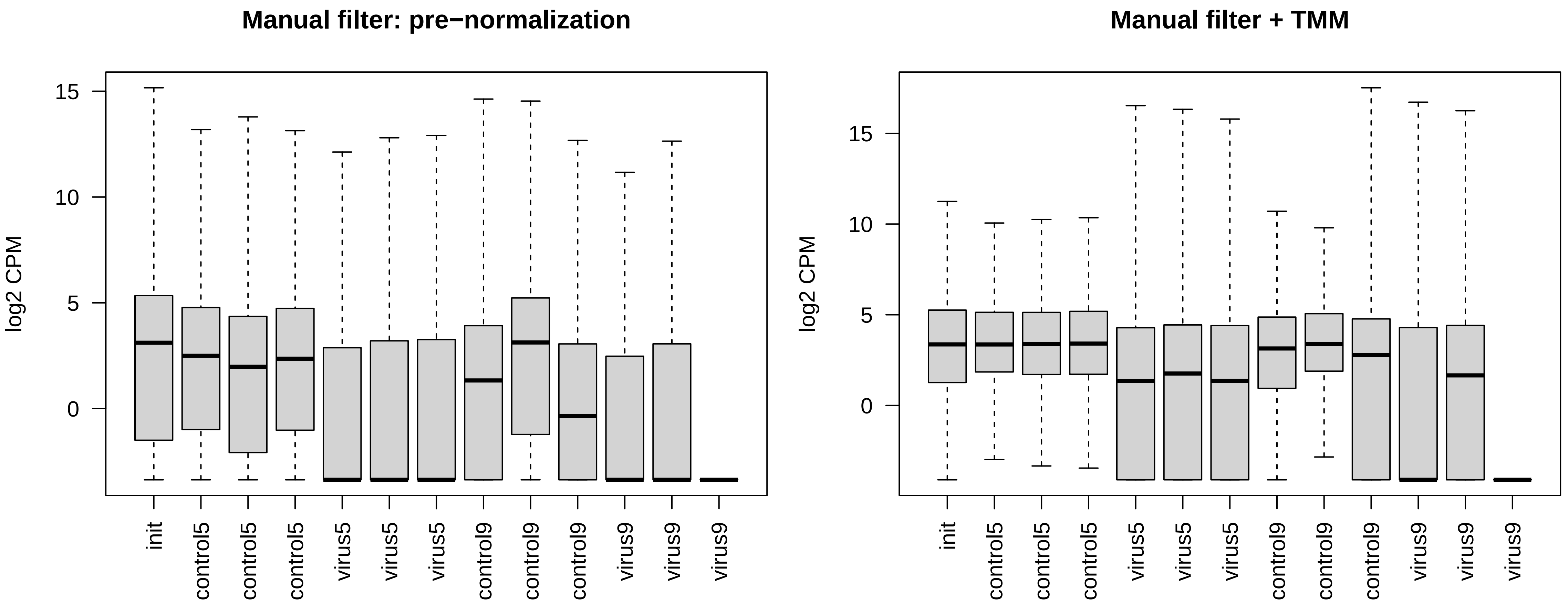
Manual filter: pre−normalization
Manual filter + TMM
log2 CPM
15
10
5
0
init
control5
control5
control5
virus5
virus5
virus5
control9
control9
control9
virus9
virus9
virus9
