# Supplementary material for: Viral lysis accelerates microbial succession patterns resembling diatom senescence
Source: ISME J. 2026 Aug 5;20(1):wrag206. doi: 10.1093/ismejo/wrag206 (PMC13590890; doi:10.1093/ismejo/wrag206)
Supplement: Supplementary file 1 [file web_material_wrag206.zip › Supplementary Figure Captions_revised - 2.docx]

**Supplementary Figure Captions:**

Figure S1. Culture flasks after 5 days of experiment. First three from left: infected cultures. Second three from left: control cultures.

Figure S2. Nutrient and DOC concentration from the start of the experiment to Day 5.

Figure S3. Bacterial species richness and Shannon Index from 16S sequencing for groups of samples. Control5 (non-infected samples after 5 days); Virus5 (infected samples after 5 days); Control9 (non-infected samples after 9 days); Virus9 (infected samples after 9 days).

Figure S4. Change of relative abundance of specific taxa over the course of the experiments inferred from 16S sequencing. A) Relative abundance of bacterial classes. B) Relative abundance of bacterial families. C) Relative abundance of 10 most abundant bacterial genera.

Figure S5. Number of microbes per diatom cell (from DAPI staining) in the A) phycosphere; B) detritosphere.

Figure S6. COG categories of DE genes for Day 9 DEA (B). The baseline for expression were control (non-infected) samples. Regulation direction is for infected samples.

Figure S7. Number of DE genes with CAZy annotation for each genus and Day. The baseline for expression were control (non-infected) samples. Regulation direction is for infected samples.

Figure S8. Analysis of coexpression between *TonB/SuSD* annotated genes and CAZymes for two most abundant genera in our cultures. A) *Polaribacter* network for Day 5 DEA. B) *Polaribacter* network for Day 9 DEA. C) *Marinobacter* network for Day 5 DEA. D) *Marinobacter* network for Day 9 DEA.

Figure S9. Heatmaps represent glucan degradation pathway expression (mean logCPM, TMM normalized). The overlayed values show relative completeness of the pathway obtained from dbCANsub (percentage of genes among all the genes found in all metatranscriptomes obtained in this study). Control5 (non-infected samples after 5 days); Infected5 (infected samples after 5 days); Control9 (non-infected samples after 9 days); Infected9 (infected samples after 9 days).

Figure S10. Boxplots of per-contig expression after filtration and TMM normalization of the diatom transcript dataset.
