# Supplementary material for: Viral lysis accelerates microbial succession patterns resembling diatom senescence
Source: ISME J. 2026 Aug 5;20(1):wrag206. doi: 10.1093/ismejo/wrag206 (PMC13590890; doi:10.1093/ismejo/wrag206)
Supplement: Supplementary file 1 [file web_material_wrag206.zip › Supplementary Method 1 - qPCR assay design.docx]

**qPCR assay design and validation**

The primers and probe were designed with Primer express 2.0 software (Applied biosystems) using as template the RdRP gene sequence of PnGalRNAV (GenBank accession OR271362). Specificity was tested *in silico* with PrimerBLAST (NCBI) with no unspecific hits identified. We used TaqMan™ Fast Virus 1-step Mastermix (Applied Biosystems) with forward and reverse primer concentration of 900nM and probe concentration of 250nM and 10 µl reaction volumes. The qPCR protocol was run as per reagent manufacturer's recommendations on a QuantStudio™ 7 Pro (Thermo Fisher Scientific). Standards and positive controls were prepared from the viral lysate that was used in the experiment. RNA was extracted from 200µl of the lysate using QIAamp MinElute Virus Spin Kit (Qiagen, Hilden) with added carrier RNA according to the manufacturer’s guidelines. 10-fold dilutions of RNA in RNAse free water were prepared until extinction. Standard dilutions were applied to the plate in pentaplicates and were used for estimation of the quantity of the target by plotting the obtained Cq values against theoretical concentrations (copies/ul) in each dilution. A theoretical concentration of 1 copy/ul was assigned to the last dilution giving a positive result in the qPCR reaction. Data was analyzed in the Design and Analysis software v. 2.8 (Thermo Fisher Scientific). We set the fluorescence threshold manually at 0.29, above the baseline and sufficiently low to be within the exponential increase region of the amplification curve. The regression line obtained from the serial dilution analysis and used as calibration curve is presented in Fig. 1 (below). The limit of quantification (LOQ) was defined as the concentration (theoretical copies/ml) of the dilution point for which the standard deviation of the Cq values within the pentaplicate was > 0.5 (this resulted to be 10^4^ copies/mL, see table below). The limit of detection (LOD) was defined as the concentration (theoretical copies/ml) of the first dilution point for which all replicates within the pentaplicate gave undermined result (which resulted to be 10^3^ copies/mL, see table below). Negative extraction as well as non-template controls, where only RNase free water was added to the extraction and to the RT-qPCR reaction, respectively, were used throughout the procedure and did not produce detectable signal. The MIQE checklist for assay accuracy is in Table 1 (below).

**Fig. 1: RT-qPCR calibration curves**

**Table 1: Calibration curve determination and LOD/LOQ calculation.**

| **Sample name** | **log copies/uL** | **Cq** | **Average** | **CqStdev** | **ΔCq** | **Copies/ul** |
| --- | --- | --- | --- | --- | --- | --- |
| PnGaLV_lysate | 7 | 15.56292138 | 15.5239 | 0.032067 |  | 10000000 |
|  | 7 | 15.50708317 |  |  |  | 10000000 |
|  | 7 | 15.51583422 |  |  |  | 10000000 |
|  | 7 | 15.47697172 |  |  |  | 10000000 |
|  | 7 | 15.55659253 |  |  |  | 10000000 |
| PnGaLV_lysate 10x | 6 | 18.74315032 | 18.6256 | 0.090408 | 3.1017459 | 1000000 |
|  | 6 | 18.5963991 |  |  |  | 1000000 |
|  | 6 | 18.68405134 |  |  |  | 1000000 |
|  | 6 | 18.62980646 |  |  |  | 1000000 |
|  | 6 | 18.47472553 |  |  |  | 1000000 |
| PnGaLV_lysate 100x | 5 | 21.72612507 | 21.7246 | 0.15077 | 3.0989488 | 100000 |
|  | 5 | 21.75345661 |  |  |  | 100000 |
|  | 5 | 21.70106373 |  |  |  | 100000 |
|  | 5 | 21.95801699 |  |  |  | 100000 |
|  | 5 | 21.48421411 |  |  |  | 100000 |
| PnGaLV_lysate 1000x | 4 | 24.84031661 | 24.9241 | 0.090124 | 3.1995005 | 10000 |
|  | 4 | 24.94837595 |  |  |  | 10000 |
|  | 4 | 24.95316052 |  |  |  | 10000 |
|  | 4 | 25.06515704 |  |  |  | 10000 |
|  | 4 | 24.81336906 |  |  |  | 10000 |
| PnGaLV_lysate 10000x | 3 | 28.22121284 | 28.2254 | 0.136028 | 3.3012957 | 1000 |
|  | 3 | 28.24676698 |  |  |  | 1000 |
|  | 3 | 28.38727521 |  |  |  | 1000 |
|  | 3 | 27.97802772 |  |  |  | 1000 |
|  | 3 | 28.29357491 |  |  |  | 1000 |
| PnGaLV_lysate 100000x | 2 | 32.2726189 | 31.6423 | 0.336671 | 3.4168832 | 100 |
|  | 2 | 31.43082014 |  |  |  | 100 |
|  | 2 | 31.5915987 |  |  |  | 100 |
|  | 2 | 31.62288099 |  |  |  | 100 |
|  | 2 | 31.29335491 |  |  |  | 100 |
| PnGaLV_lysate 1000000x | 2 | 35.19324005 | 35.1866 | 1.017558 | 3.5443161 | 100 |
|  | 1 | 35.81422599 |  |  |  | 10 |
|  | 1 | 33.93984163 |  |  |  | 10 |
|  | 1 | 34.25963224 |  |  |  | 10 |
|  | 1 | 36.72591424 |  |  |  | 10 |
| PnGaLV_lysate 10000000x |  | Undetermined |  | / | / | 1 |
|  |  | Undetermined |  |  |  | 1 |
|  |  | Undetermined |  |  |  | 1 |
|  |  | Undetermined |  |  |  | 1 |
|  |  | Undetermined |  |  |  | 1 |
| PnGaLV_lysate 100000000x |  | Undetermined | 38.767 | / | / |  |
|  |  | Undetermined |  |  |  |  |
|  |  | 38.76698838 |  |  |  |  |
|  |  | Undetermined |  |  |  |  |
|  |  | Undetermined |  |  |  |  |
| PnGaLV_lysate 1000000000x |  | Undetermined |  | / | / |  |
|  |  | Undetermined |  |  |  |  |
|  |  | Undetermined |  |  |  |  |
|  |  | Undetermined |  |  |  |  |
|  |  | Undetermined |  |  |  |  |

**Table 2: (MIQE Checklist)**

| **ITEM TO CHECK** | **IMPORTANCE** | **CHECKLIST** | **COMMENTS/WHERE?** |
| --- | --- | --- | --- |
| **EXPERIMENTAL DESIGN** |  |  |  |
| Definition of experimental and control groups | **E** | **YES** | Supplementary Methods |
| Number within each group | **E** | **YES** | Supplementary Methods |
| Assay carried out by core lab or investigator's lab? | D | YES | / |
| Acknowledgement of authors' contributions | D | YES | Acknowledgements |
| **SAMPLE** |  |  |  |
| Description | **E** | YES | Supplementary Methods |
| Volume/mass of sample processed | D | 200ul | Supplementary Methods |
| Microdissection or macrodissection | **E** | NA |  |
| Processing procedure | **E** | YES | Methods, Supplementary Methods |
| If frozen - how and how quickly? | **E** | YES | -80°C, immediately after sampling |
| If fixed - with what, how quickly? | **E** | NA | / |
| Sample storage conditions and duration (especially for FFPE samples) | **E** | YES | -20°C, 1 year |
| **NUCLEIC ACID EXTRACTION** |  |  |  |
| Procedure and/or instrumentation | **E** | YES | Supplementary Methods |
| Name of kit and details of any modifications | **E** | YES | Supplementary Methods |
| Source of additional reagents used | D | NA | / |
| Details of DNase or RNAse treatment | **E** | YES | No treatment |
| Contamination assessment (DNA or RNA) | **E** | YES | Negative controls of isolation |
| Nucleic acid quantification | **E** | YES | Qubit |
| Instrument and method | **E** | YES | Supplementary Methods |
| Purity (A260/A280) | D | YES | Bellow detection |
| Yield | D | YES | Bellow detection |
| RNA integrity method/instrument | **E** | YES | Tapestation |
| RIN/RQI or Cq of 3' and 5' transcripts | **E** | NA |  |
| Electrophoresis traces | D | NA |  |
| Inhibition testing (Cq dilutions, spike or other) | **E** | Yes | Cq dilutions |
| **qPCR TARGET INFORMATION** |  |  |  |
| If multiplex, efficiency and LOD of each assay. | **E** | NA |  |
| Sequence accession number | **E** | YES | Results |
| Location of amplicon | D | YES | Methods, Supplementary Methods |
| Amplicon length | **E** | YES | Methods |
| *In silico* specificity screen (BLAST, etc) | **E** | YES | PrimerBLAST, BLAST |
| Pseudogenes, retropseudogenes or other homologs? | D | NA |  |
| Sequence alignment | D | NA |  |
| Secondary structure analysis of amplicon | D | YES | OligoAnalyzer 3.1 |
| Location of each primer by exon or intron (if applicable) | **E** | YES | EXON |
| What splice variants are targeted? | **E** | NA |  |
| **qPCR OLIGONUCLEOTIDES** |  |  |  |
| Primer sequences | **E** | YES | Methods |
| RTPrimerDB Identification Number | D | NA |  |
| Probe sequences | D** | YES | Methods |
| Location and identity of any modifications | **E** | NA |  |
| Manufacturer of oligonucleotides | D | YES | Eurofins Genomics |
| Purification method | D | YES | HPLC |
| **qPCR PROTOCOL** |  |  |  |
| Complete reaction conditions | **E** | YES | Supplementary Methods |
| Reaction volume and amount of cDNA/DNA | **E** | YES | Supplementary Methods |
| Primer, (probe), Mg++ and dNTP concentrations | **E** | YES | Supplementary Methods |
| Polymerase identity and concentration | **E** | YES | Supplementary Methods |
| Buffer/kit identity and manufacturer | **E** | YES | Supplementary Methods |
| Exact chemical constitution of the buffer | D | NO | Manufacture proprietary |
| Additives (SYBR Green I, DMSO, etc.) | **E** | NA |  |
| Manufacturer of plates/tubes and catalog number | D | NA |  |
| Complete thermocycling parameters | **E** | YES | Manufacturer's guidelines |
| Reaction setup (manual/robotic) | D | YES | Manual |
| Manufacturer of qPCR instrument | **E** | Yes | Supplementary Methods |
| **qPCR VALIDATION** |  |  |  |
| Evidence of optimisation (from gradients) | D | NO |  |
| Specificity (gel, sequence, melt, or digest) | **E** | YES | *In silico,* supplementary |
| For SYBR Green I, Cq of the NTC | **E** | NA |  |
| Standard curves with slope and y-intercept | **E** | YES | Supplementary Methods, Fig.1 |
| PCR efficiency calculated from slope | **E** | YES | Supplementary Methods, Fig.1 |
| Confidence interval for PCR efficiency or standard error | D | NO |  |
| r2 of standard curve | **E** | YES | Supplementary Methods, Fig.1 |
| Linear dynamic range | **E** | YES | Supplementary Methods, Fig.1 |
