# Supplementary material for: Viral lysis accelerates microbial succession patterns resembling diatom senescence
Source: ISME J. 2026 Aug 5;20(1):wrag206. doi: 10.1093/ismejo/wrag206 (PMC13590890; doi:10.1093/ismejo/wrag206)
Supplement: Supplementary file 1 [file web_material_wrag206.zip › Supplementary Methods 2- Metatranscriptomic pipeline_review.docx]

***ALDEx2 analysis of bacterial metatranscriptomes***

*ALDEx2* (v1.30.0) [1] was used to identify differentially expressed bacterial genes between treatments. salmon-derived transcript counts were extracted and filtered to include only bacterial transcripts with more than 10 reads and present in at least 4 samples. Centered log-ratio (CLR) transformation was performed using aldex.clr. Differential abundance testing was conducted using Welch’s t-test (aldex.ttest) with Benjamini–Hochberg FDR correction. Genes with effect sizes >1 and adjusted *p*-values (*eBH*) < 0.1 were considered significantly differentially expressed., Salmon-derived transcript counts were extracted and filtered to include only bacterial transcripts, and centered log-ratio (CLR) transformation was performed using aldex.clr in the ALDEx2 R package [1]. Differential abundance testing was conducted using Welch’s t-test (aldex.ttest) with Benjamini–Hochberg FDR correction. Genes with effect sizes >1 and adjusted *p*-values (*eBH*) < 0.1 were considered significantly differentially expressed. Quality check of the analysis was performed using volcano plots, which demonstrated likely very high false negative results and thus the analysis was not carried forward, as *edgeR* offered more balanced results with more statistical confidence.

***DESeq2 analysis of host transcriptomes***

Due to heavily unbalanced datasets, ALDEx2 was unsatisfactory for the diatom dataset as it produced only non-significant results. We thus tested the data with *DESeq2* as well. Variance-stabilized expression values were obtained from the full host transcriptome by applying the DESeq2 vst() transformation to filtered count data (genes with >10 counts in at least 4 samples). This analysis resulted in many significant DE genes (>1000) but with a clearly high false positive rate. Comparison with the *edgeR* analysis confirmed that all the *edgeR* genes were in the *DESeq2* dataset. *edgeR* was thus chosen as the most stable and robust method for our analysis also for the host transcriptomes.

***Additional annotation with AlphaFold2***

To gain functional insight into DE transcripts with high log2FC lacking annotation in the eggNOG-mapper pipeline, we submitted unannotated but significantly differentially expressed sequences to *AlphaFold2* via *ColabFold* [2] and performed structure-based homology searches using *FoldSeek* [3] against the AlphaFoldDB. Hits were manually curated and used to update functional annotations of previously uncharacterized genes. These were integrated into downstream analyses, including pathway enrichment and genus-function association visualizations.

***Analysis of CAZymes***

We curated a set of marker CAZy families [4] and PUL-associated genes representative of seven polysaccharide substrates or substrate analogues that are produced by diatoms: β-glucan (laminarin), alginate, chitin, xylan, β -mannan, exopolysaccharide and host-glycan. Genes and gene families associated with degradation of these substrates were sourced from the dbCANsub database. Relative pathway completeness per sample was calculated as the proportion of these marker families detected (at least one contig with CPM > 0.1), divided by the total number of families found in our data across all samples for each substrate (Supplementary Table 9). We are aware this is less conservative estimate than using the entire set of known CAZy families but it is unlikely that a selected set of bacteria present in the cultures would possess all the different degradation enzymes, while we did not have metagenomic data to estimate this. We acknowledge that this is a minimalist marker set, and additional CAZy families (e.g., auxiliary debranching enzymes, additional CBMs, or alternative lyases) likely contribute to full pathway function. However, this conservative definition offers a standardized and interpretable framework for comparing pathway erosion or expansion across treatments.

To explore coordinated expression between carbohydrate-active enzymes and substrate uptake systems, we constructed genus-resolved CAZyme–TonB coexpression networks for two subsets of the bacterial metatranscriptome, corresponding to transcripts annotated to *Polaribacter* and *Marinobacter*. Because CAZy family assignments obtained through eggNOG were limited in resolution for this purpose, the functional annotation tables were expanded with dbCAN-derived CAZy annotations. For each transcript, one best-supported dbCAN annotation was retained after collapsing protein isoforms to the transcript level, and only reannotated CAZy assignments supported by at least two dbCAN tools were accepted. Candidate CAZyme transcripts were defined as those carrying either an eggNOG-derived CAZy annotation or a retained dbCAN-based CAZy reassignment. Candidate TonB-related transcripts were identified from annotation fields (preferred name, description, and PFAMs) using keywords corresponding to TonB-dependent receptors and SusC/RagA-like outer membrane transporters. Pairwise coexpression was then evaluated separately within the selected bacterial genera by calculating Spearman correlations between all CAZyme and TonB-candidate transcript pairs across samples. Only within-genus CAZyme–TonB pairs were considered, thereby restricting the analysis to genes assigned to the same taxonomic group. Resulting correlations were corrected for multiple testing within each genus using the Benjamini–Hochberg procedure, and significant positive associations were retained for network visualization.

**References cited**

1.	Fernandes AD, Macklaim JM, Linn TG *et al.* ANOVA-like differential expression (ALDEx) analysis for mixed population RNA-Seq. *PLoS One*. 2013;**8**:e67019 <https://doi.org/10.1371/journal.pone.0067019>

2.	Mirdita M, Schutze K, Moriwaki Y *et al.* ColabFold: making protein folding accessible to all. *Nat Methods*. 2022;**19**:679-82 <https://doi.org/10.1038/s41592-022-01488-1>

3.	van Kempen M, Kim SS, Tumescheit C *et al.* Fast and accurate protein structure search with Foldseek. *bioRxiv*. 2023 <https://doi.org/10.1101/2022.02.07.479398>

4.	Cantarel BL, Coutinho PM, Rancurel C *et al.* The Carbohydrate-Active EnZymes database (CAZy): an expert resource for Glycogenomics. *Nucleic Acids Res*. 2009;**37**:D233-8 <https://doi.org/10.1093/nar/gkn663>
