# Supplementary material for: Viral lysis accelerates microbial succession patterns resembling diatom senescence
Source: ISME J. 2026 Aug 5;20(1):wrag206. doi: 10.1093/ismejo/wrag206 (PMC13590890; doi:10.1093/ismejo/wrag206)
Supplement: Supplementary file 1 [file web_material_wrag206.zip › Supplementary Table 10.docx]

**Table S9: Quantitative and qualitative description of aggregates at different stages of the experiment**

| **ID_sample** | **NUMBER OF AGGREGATES BY SIZE** | | | **NOTES** | **FIGURES** |
| --- | --- | --- | --- | --- | --- |
|  | small | medium | large |  |  |
| D1C1 | 14 | 8 |  | Aggregates observed in the sample contain diatoms. | Figure1 |
| D1C2 | 9 | 6 | 1 | Aggregates observed in the sample contain diatoms. | Figure2 |
| D1C3 | 17 |  |  | Majority of the aggregates observed in the sample are small and contain diatoms. | Figure3 |
| D1V1 | 16 | 2 |  | Majority of the aggregates observed in the sample contain diatoms. | Figure4 |
| D1V2 | 8 | 5 |  | Majority of the aggregates observed in the sample contain diatoms. | Figure5 |
| D1V3 | 13 | 5 |  | High abundance of aggregates observed in the sample; majority of the aggregates contain diatoms. | Figure6 |
| D3C1 | 6 | 5 |  | Some aggregates observed in the sample contain more diatoms, while other contain fewer. | Figure7  Figure8 |
| D3C2 | 17 | 2 |  | Majority of the aggregates observed in the sample contain a higher abundance of diatoms. | Figure9 |
| D3C3 | 6 |  | 1 | Some aggregates observed in the sample contain more diatoms, while other contain fewer. | Figure10 |
| D3V1 | 14 |  | 7 | Some aggregates observed in the sample contain more diatoms, while other contain fewer; a few non-compact aggregates were observed. | Figure11  Figure12 |
| D3V2 | 9 | 9 |  | Aggregates observed in the sample contain some diatoms; a few non-compact aggregates were observed. | Figure13  Figure14 |
| D3V3 | 9 | 8 | 5 | Aggregates observed in the sample contain some diatoms; a few non-compact aggregates were observed. | Figure15 |
| D5C1 | 16 | 9 |  | Majority of the aggregates observed in the sample contain diatoms; a few non-compact aggregates were observed. | Figure16 |
| D5C2 | 23 | 7 | 2 | Majority of the aggregates observed in the sample contain diatoms; a few non-compact aggregates were observed. | Figure17 |
| D5C3 | 12 | 23 | 2 | Aggregates observed in the sample contain diatoms | Figure18 |
| D5V1 | 4 | 4 | 8 | Aggregates observed in the sample contain diatoms; majority of the aggregates observed were non-compact. | Figure19 |
| D5V2 | 5 |  | 2 | Majority of the aggregates observed in the sample contain few diatoms; majority of the aggregates observed were non-compact. | Figure20  Figure21 |
| D5V3 |  | 1 |  | Majority of the aggregates observed in the sample contain few diatoms; majority of the aggregates observed were non-compact. | Figure22  Figure23 |
| D9C1 | 2 |  | 1 | Majority of the aggregates observed in the sample were small and some contained diatoms. | Figure24  Figure25 |
| D9C2 | 2 | 2 | 1 | Some of the aggregates contained diatoms; some of the aggregates were non-compact. | Figure26 |
| D9C3 | 9 | 5 | 1 | Some of the aggregates contained diatoms; some of the aggregates were non-compact. | Figure27 |
| D9V1 | 1 |  | 2 | Majority of the aggregates observed in the sample were non-compact. | Figure28 |
| D9V2 |  | 3 | 2 | Majority of the aggregates observed in the sample were non-compact. Some of the aggregates have diatoms. | Figure29 |
| D9V3 |  |  |  | Aggregates observed in the sample were non-compact. | Figure30 |

| DAY | CONTROL | VIRUS |
| --- | --- | --- |
| 1 |   Figure 1    Figure 2    Figure 3 |   Figure 4    Figure 5    Figure 6 |
| 3 |   Figure 7    Figure 8    Figure 9    Figure 10 |   Figure 11    Figure 12    Figure 13    Figure 14    Figure 15 |
| 5 |   Figure 16    Figure 17    Figure 18 |   Figure 19    Figure 20    Figure 21    Figure 22    Figure 23 |
| 9 |   Figure 24    Figure 25    Figure 26    Figure 27 |   Figure 28    Figure 29    Figure 30 |
